# Australian rural service learning student placements: a national survey

**DOI:** 10.1186/s12909-024-05172-0

**Published:** 2024-03-01

**Authors:** Monica Moran, Sarah Miles, Priya Martin

**Affiliations:** 1 Western Australian Centre for Rural Health (WACRH), 167, Fitzgerald St, Geraldton, WA 6530 Australia; 2 University Centre for Rural Health, University of Sydney Lismore, Lismore, NSW 2480 Australia; 3 Rural Clinical School, Faculty of Medicine, The University of Queensland, Locked Bag 9009, Toowoomba, QLD 4350 Australia

**Keywords:** Service learning, Placements, Rural health, Rural and remote

## Abstract

This preliminary national study is the first of its kind to investigate how service learning placements are implemented in real world settings in rural Australia and what factors enable or hinder their implementation. An anonymous survey was distributed to 17 University Departments of Rural Health (UDRH) in Australia. Numerical data were analysed descriptively. Textual data were analysed using a hybrid content analysis approach. Thirty seven respondents provided data representing 12 UDRHs. Responding UDRHs reported facilitating service learning programs, with experience in this context ranging from 3 months to 21 years. Service learning placements predominantly occurred in schools and aged care facilities. Occupational therapy, physiotherapy, and speech pathology were the most frequently involved professions in service learning. Enablers and barriers identified were categorised into: People, Partnerships, and Place and Space. This national-scale study provides a springboard for more in-depth investigation and implementation research focused on development of a conceptual model to support service learning across rural and remote Australia.

**Supplementary Information:**

The online version contains supplementary material available at 10.1186/s12909-024-05172-0.

## Introduction

Service learning is a pedagogical approach often seen as a subset of work integrated learning (WIL). Evolving in university programs in North America, the focus was on students predominantly completing community activities, civil engagement, and social justice projects, in order to build good citizenship skills beyond those developed in discipline-specific silos [1]. In Australian universities the term has more recently transitioned to reference a range of WIL activities that provide students with work exposure in vocational environments where they utilize the professional skills they are developing as part of their academic programs. One form of service learning is where healthcare students are supported to complete placements providing direct clinical or professional services where there are limited to no services. This may also be referred to as a student-led, student-assisted, or student-implemented service [2]. Such service learning approaches balance student learning and the healthcare needs of the community in which they are implemented [3]. In other words, there is equal weighting between student learning and service outcomes [4, 5]. Service learning programs are more common in resource-constrained rural and remote contexts in countries such as Australia that experience health workforce recruitment and retention issues [3]. Examples are also available of service learning programs embedding interprofessional education (IPE) principles within them. For example, a rural New South Wales study explored occupational therapy and speech pathology students’ and supporting academics’ experiences of being involved in the Allied Health in Outback Schools Program which incorporated IPE elements [3].

Routinely, service learning programs are embedded within the community including schools, aged care facilities, and home and community organisations [2]. Previous research has highlighted the important role communities play in supporting students while they are delivering the service learning programs [6]. For example, strong ties with communities meant that service learning placements could continue in remote Northern Australia even through the COVID-19 pandemic, utilising telehealth models [2].

Given the large landmass in Australia, there are significant variations between and within regional, rural, and remote areas. The Monash Modified (MM) Model developed by the Australian Department of Health is a widely used remoteness classification system to identify the disparities in access to health services across Australia [7]. It is structured into seven remoteness categories (1–7) with MM1 representing major cities and metropolitan areas, MM2 representing regional centres, MM3 representing large rural towns, MM4 representing medium rural towns, MM5 representing small rural towns, MM6 representing remote communities, and MM7 representing very remote communities. Approximately 30% of Australians reside in areas that are geographically classified as regional, rural, and remote (MM categories 2–7) [7].

Populations in these non-metropolitan areas are known to have unique challenges due to their geographic isolation. Regional, rural, and remote communities have higher rates of hospitalizations, mortality, injury, and decreased access to and the usage of health care services [8]. The Australian Government Department of Health and Aged Care is committed to improving rural health outcomes and has developed the Stronger Rural Health Strategy to improve the health of people living in rural Australia [9].

One of the responses to the Stronger Rural Health Strategy is the Rural Health Multidisciplinary training (RHMT) program. This program funds a network of 17 University Departments of Rural Health (UDRH) across Australia. UDRHs are tasked with undertaking a range of activities including rural health research, and the provision of quality health placements for nursing, and allied health students [10]. The strategic direction of these programs is grounded in the growing body of literature demonstrating that students who either grow up in rural areas or are exposed to quality rural placements are more likely to come back to practice rurally [11, 12]. UDRHs have a strong focus on population health across rural Australia, particularly in MM 2 to 7 locations [7]. Additionally with the expansion of tertiary programs for allied health professions and the current movement in workforce into the private and community sectors, UDRHs strive to create innovative placements that expand beyond the more traditional hospital/clinic locations. Service learning is identified by UDRHs as a way to add value to areas of need, meet the RHMT requirements, and also provide students with high quality placement experiences.

The Australian Rural Health Education Network (ARHEN) is the national association and peak body for the UDRHs [13]. The ARHEN network supports a number of special interest membership groups including a Service Learning Group. This group is made up of representatives from the majority of UDRHs who are interested in or actively offer service learning educational experiences. Despite the prevalence of service learning programs across the rural and remote locations serviced by UDRHs in Australia there is little known about the similarities and differences, program priorities, governance arrangements, and community collaborations across sites. There is no agreed overarching process for the implementation of service learning.

Given the limited understanding of the scope and range of service learning activities across the ARHEN network, members of the Service Learning Group developed a national survey to investigate how service learning is being enacted across rural and remote Australia through the UDRHs and what factors enabled or hindered the utilization of such programs. The ARHEN board of directors agreed to provide a small grant to support the recruitment of an independent research officer to manage the project. This helped minimise any conflict of interest between the service learning network and UDRHs.

Internationally, clinical education frameworks have been reported in the literature to play a role in articulating service learning models. The Best Practice Clinical Learning Environment Framework (BPCLE) focuses on delivering quality clinical education for learners and considers both academic and industry factors crucial to delivering high quality clinical education. It describes six elements namely organisational culture, best practice clinical practice, positive learning environment, effective health service-education provider partnership, effective communication processes, and appropriate resources and facilities [14]. This framework, although used widely, does not consider community factors that are a key part of service learning. The community-based medical education (CBME) framework proposed by Kelly and colleagues [15] in the context of medical education in primary care, highlights the role of suitable preceptors or supervisors, community, and time during community placement for students in developing relationships with supervisors and service users that are meaningful, thereby leading to positive learning experiences. This model considers student, supervisor, as well as community factors in contributing to positive learning outcomes. Collectively, elements from both the BPCLE and CBME frameworks can be useful while examining service learning.

This research project set out to both inductively and deductively examine the implementation of service learning across the UDRHs in rural Australia guided by available international educational frameworks.

## Methods

### Participants

Participants were academics, administrators, or clinical educators (student supervisors) of participating UDRHs. Each participating UDRH was asked to submit no more than one response, and more than one person at each UDRH was able to input into the survey.

### Materials and procedure

A brief, anonymous mixed methods survey was developed and administered online through REDcap. Questions related to the service learning placements used by the UDRH, terminologies these models were known by, professions involved, duration of use of such models, community settings that were utilised, availability of governance structures, evaluation of such models, and enablers and barriers to the implementation of these models. Questions elicited a mix of closed (i.e., categorical) and open-ended (i.e., free text) responses. The survey was developed collaboratively and iteratively over a one year period by members of the ARHEN Service Learning Group. Survey design and item development were informed by Patton [16] with a focus on congruence between the qualitative items that explored the experience of delivering service learning programs, and quantitative items that illuminated frequency, ranges, and involvement of students from specific professions. Peer and expert feedback was sought and provided by the ARHEN directors. The draft survey was piloted with five people, all with experience in service learning, and revised for readability and accessibility. The link to the online survey, along with a participant information form, was distributed by the National Director of ARHEN (external to the research team) for dissemination to UDRH directors who facilitated completion of the survey within their organisations. Three reminders were sent by the ARHEN Director to boost the response rate. Participants provided consent online prior to completing the survey. The survey was open between August and October 2022. The survey has been included as supporting information.

### Data analysis

Data were extracted from REDcap and fully anonymised prior to analysis. Numerical data were analysed descriptively. Textual data from the free text responses on enablers and barriers to the implementation of service learning were analysed using a hybrid content analysis approach by two researchers (MM, PM) in the team with experience in clinical education, rural health, mixed methods and qualitative research, service learning, and a background in occupational therapy. As a first step a Priori codes were developed based on elements of the BPCLE and CBME frameworks, facilitating deductive analysis. Onto this, inductive analysis was overlaid to enable category development from the data [17, 18]. This hybrid approach enabled close alignment with theory, while still being open to meaning arising from the dataset. Further information about the development of codes and categories is presented in Table 1. The second author (SM) verified the categories after becoming familiar with the de-identified dataset and also contributed to the interpretation of findings. Only the last author (PM), who is not affiliated with ARHEN accessed identifiable data. The first and second authors (MM, SM), with current UDRH affiliations, only had access to de-identified data and remained separate to the survey distribution and completion processes to minimise potential bias.

**Table 1:** Development of codes to inform categories

Framework	Elements Used	Final Categories
CBME (Kelly et al., 2014)	Patient/consumer	People
	Supervisor	
	Student	
	Relationships	Partnerships
BPCLE (DHHS, 2016)	Health service-Education provider partnership	
	Communication processes	
	Learning environment	Place and space
	Resources and facilities	

### Ethics

Ethics approval for this study was obtained from the University of Western Australia Human Research Ethics Committee (Ref: ET000454; approval date: 01/08/2022).

## Results

Thirty seven respondents provided data through 13 survey responses representing 12 UDRHs (UDRH response rate = 76.5%). Twelve responding UDRHs reported to facilitating service learning programs, with experience in this context ranging from 3 months to 21 years. Across the twelve UDRHs facilitating service learning, the most commonly used terminology was ‘service learning’ (*n* = 11, 91.7%), followed by ‘project placements’ (n = 8, 66.7%). All UDRHs offered service learning placement opportunities delivered in partnership with existing organisations such as schools and aged care facilities (n = 12, 100%), as well as project based (n = 12, 100%). Additionally, over half of UDRHs (50–58%), facilitated service learning placements as outreach services, in student-led clinics, and in Aboriginal Community Controlled Health Organisations.

Respondents described using a variety of supervision models within service learning including discipline-specific, interprofessional, long arm, peer supervision, and telesupervision models. Occupational therapy, physiotherapy, and speech pathology (*n* = 11, 91.7% each) were the most frequently involved professions in service learning. Most placements were located in remote MM6 sites (*n* = 39), followed by very remote MM7 sites (*n* = 37). Most respondents (*n* = 9, 75%) reported to having governance structures in place for the service learning placements. All respondents (*n* = 12, 100%) reported to researching or evaluating the service learning programs employed in their UDRH, with eight of those sites involved in or intending to be involved in publishing results from the research or evaluation. Further characteristics of service learning placements reported by respondents can be found in Table 2.

**Table 2:** Characteristics of service learning placements across Twelve UDRHs

Variable	Categories	N, %	Further Information
Terms used	Service learning Project placements Work-ready Role emerging Other	11, 92 8, 67 5, 42 2, 17 3, 25	Other: Complex health and/or learning initiatives/programs/strategies. Extended duration/immersive placements.
Placement sites	Organisational based (Schools, aged care facilities etc.) Project-based Outreach service Student-led clinic Aboriginal Community controlled Health Organisations (ACCHOS) Research-based Other	12, 100 12, 100 7, 58 6, 50 6, 50 5, 42 4, 34	Other: Mental Health and Disability focused services
Discipline	Physiotherapy Speech pathology Occupational therapy Nutrition and Dietetics Social Work Exercise Physiology Nursing Audiology Oral Health Chiropractic Dentistry Music Therapy Podiatry Medical Imaging Optometry Other	11, 92 11, 92 11, 92 8, 67 8, 67 5, 42 4, 33 3, 25 2, 17 1, 8 1, 8 1, 8 0. 0 0, 0 0. 0 6, 50	Other: Exercise Science (*n* = 2) Pharmacy (*n* = 3) Health Science (*n* = 1) Psychology (*n* = 1) Osteopathy (*n* = 1)
Placement site location	MM2 MM3 MM4 MM5 MM6 MM7	15 42 24 19 39 37	
Governance structure in place	Yes No	9, 75 5, 42	
Research/evaluation of service learning	Yes No	12, 100 2, 17	
Publication if researching	Yes No	8, 67 4, 33	

### Enablers of and barriers to service learning placement models

Three categories were developed including People, Partnerships, and Place and Space. The ‘People’ category includes key stakeholders such as the student, student supervisor (or clinical educator) who is usually an UDRH staff member, parent university staff (usually more centrally located) and client or service users in the community. The ‘Partnership’ category reflects relationships between key stakeholders and the integral role of supervision and support. The ‘Place and Space’ category reflects the resources and infrastructure required for service learning programs and the role of remoteness and isolation in this context. The impact of COVID-19 was captured in this category as maintaining student placements ‘in place’ or in alternate spaces. Under each category, enablers, barriers, and recommendations where available, have been encapsulated to enable a seamless flow to aid interpretation.

## 1. People

### Student

Respondents described the ideal student for successful service learning placements to be one with a genuine interest in rural health, intention for future rural practice, flexible, prepared, open to new experience, in later years of study, doing well academically, and being able to seek support and connection. Respondents noted:

*Student having a commitment to social accountability, willingness to be open and learn, flexibility, tolerance, and adaptability to uncertainty (i.e., enablers). (Respondent 7)*

*Allocation of students to the placement who have no interest/intent to practice rural post- registration (i.e., barrier). (Respondent 13)*

*Student-led clinic (is) not a placement appropriate for first placement or (a) struggling student (i.e., barrier). (Respondent 9)*

### Placement supervisor at the UDRH

Respondents noted that implementing service learning placements is a big commitment from the UDRH staff. For successful placements those in direct supervision roles are expected to be supportive, provide good supervision, be responsive to student and community needs, and create high quality learning opportunities for students. One respondent described a significant challenge supervisors face:

*Differing placement start dates and provision of handover information (e.g., having to facilitate multiple orientation sessions and handover process requiring ongoing update and modification due to heavy student turn over). (Respondent 13)*

Another respondent noted the key role of appropriately skilled supervisors in the success of service learning placements:

*Skilled supervisors who are able to facilitate and manage a range of supervision approaches. (Respondent 12)*

### Parent university staff

Respondents commented on the lack of understanding and support from their parent university staff who are more centrally located:

Challenges in engaging home university academics/coordinators when placement/student challenges arise. (Respondent 13)

They attributed this to a lack of understanding of the value of service learning programs:

Some university staff are unfamiliar with this model and hesitant to engage. (Respondent 1)

Another respondent went on to offer a solution to mitigate this challenge. They advocated for the parent university staff involved in student placement co-ordination roles to visit rural communities where service learning programs are implemented:

*When university teams do visit remote and/or service learning sites their commitment to preparing and sending students markedly improves. Opportunities for university placement teams and academics to visit these sites need to be prioritised. (Respondent 12)*

### Consumers and community

Most service learning placements are situated in small, rural, and remote communities. Respondents described the challenges faced by consumers/service users in these communities, which can be exacerbated by withdrawing a service due to students being unavailable to sustain the service learning placements. Another challenge is the frequent changeover of students especially linked to short placement durations. Respondents noted:

*Constant rotation of new students. Don’t know what they (community) can ask for/what allied health can provide (full scope of services). Don’t know how services can be best provided to meet their needs. (Respondent 7)*

*Communities may have unrealistic expectations regarding the scope or availability of students to deliver services in rural/remote settings as part of a service learning environment. Continuous briefing and engagement with community representatives is vital to ensure that their expectations match what is possible. (Respondent 12)*

Highlighting the cultural challenges, one respondent noted that service learning placements are successful in communities that have *‘acceptance of white people and services’ (Respondent 7).*

This response reflects the importance of students having comprehensive cultural sensitivity training and the challenges of entering remote communities where there may be elements of distrust towards mainstream services and a long history of intergenerational trauma following Australian colonization.

## 2. Partnerships

Supervision and support, and flexibility from all involved stakeholders were noted to be essential elements in developing and maintaining partnerships between students, supervisors, consumers, communities, UDRHs, and parent universities. Respondents noted that communities are keen to partner with UDRHs formally and informally in order to access services that may not be otherwise available in those regions:

*Communities see themselves in partnership with (the) UDRH and want to access student services that would not otherwise be available. (Respondent 12)*

As was also noted in the previous category, partnerships with community stakeholders can be negatively impacted by the UDRH model of hosting students for limited periods of time across the year. Negotiated partnerships with clear expectations are vital to ensure that the UDRH can function within communities. One respondent noted the key role supervisors and students can play in partnering with community organisations and members to develop intentional and explicit expectations of what is possible within the partnership and how services can be negotiated with community stakeholders to ensure that their needs are valued and respected:

*The revolving door of students delivering service learning placements can place a big emotional load on communities, with short term relationships and constant goodbyes - this particularly impacts on the children. Supervisors and students need to be mindful of this issue and be very honest about their role and types of engagement in community. (Respondent 12)*

Respondents noted the need for good partnerships between UDRHs and the parent universities to support service learning. Promoting partnerships between the UDRH and parent university staff was seen as a way of addressing barriers such as rigid placement calendars, staff leave, turnover, and rigidity about student placement structures and models.

Respondents also noted that supervisors can instill in students a sense of belonging and connection while on placement. Respondents recommended partnerships across placement settings to address the lack of availability of discipline-specific supervisors:

*Discipline specific supervision needs for some placements (can be met by) part-time placements across two settings i.e., service learning plus other health care setting, so supervision is adequately covered. (Respondent 4)*

## 3. Place and space

Largely reflective of the systemic challenges faced by small rural and remote communities, resources and infrastructure issues and needs, and social isolation challenges stemming from the remoteness of these communities were highlighted in the data. The impact of the COVID-19 pandemic onset on these placements were also highlighted. Finances, infrastructure, travel costs, staffing and other human resources, accommodation costs and availability were all noted to be barriers that are commonly experienced disproportionately by UDRH organisations trying to operate in rural and remote areas. The need for targeted funding schemes, scholarships, student accommodation, and resources were unanimously agreed upon by respondents. Barriers that are place-specific such as lack of public transport and isolation associated with large remote spaces were highlighted.

Respondents noted these barriers:

*Ongoing issues of staffing in MM4-5 (Respondent 4).*

*Access to infrastructure - e.g., accommodation and vehicles- availability and cost. Recruitment of supervisors. Logistics of transporting a supporting student in remote communities. (Respondent 7)*

*Students not having a car to bring to placement or driver’s licence limits placement opportunities for the student and is isolating on weekends. (Respondent 8)*

Some respondents noted the higher costs involved in the service learning programs, and others noted the impact of the above-noted challenges on sustainability of service learning placements:

*Service learning is an expensive model, particularly in more remote communities. Advocating for funding from additional avenues is important but also tapping into local funding sources such as big industries. (Respondent 12)*

*Funding is always an issue. Without it we wouldn’t get many supervisors, but funding placements can make service learning unsustainable. We try to partner with organizations who employ the supervisors or look at things like grants, PHN (Primary Health Network) funding, etc. Sustainability is probably our biggest issue all round. Health professionals constantly moving/leaving makes it hard to continue good opportunities. (Respondent 1)*

Although the survey did not have explicit questions related to the COVID-19 pandemic, respondents discussed the impact the pandemic had on the provision of service learning placements in rural and remote communities. Students already in rural communities had to be managed in the light of changing placement structures, and students planning to travel to remote communities had to be supported to complete alterative placement experiences that still offered a service to remote communities. Respondents mentioned some mitigating strategies they used to manage and maintain service learning placements during this time:

*Impact of COVID-19 on accessing placements in usual timeframes due to student/staff availability. Some impact on types of activities able to be undertaken on placement due to COVID-19. Availability of supervisors due to isolation requirements, some reliance on virtual contact. Issues still ongoing and managed through flexible working arrangements and making up time for missed days. (Respondent 4)*

*Students (were) affected by COVID-19. Strategies to assist students affected by isolation periods has been ‘working from home’ or extending placement block to make up for missed days. (Respondent 5)*

## Discussion

This is the first known national-scale study utilising data from across Australia to investigate how service learning programs are being used across rural and remote Australia and to identify factors that enabled or hindered their utilization. By using existing international frameworks such as the CBME [15] and BPCLE [14] to inform data analysis and interpretation of findings, this study adds important evidence to a sparsely researched field. While both frameworks are well-established in their own right, neither one fully accounted for all aspects of the service learning programs operated by UDRHs in rural and remote Australia. While elements of the CBME framework emphasize on stakeholders (i.e., patients, supervisors, and students) and relationships, elements of the BPCLE framework focus on partnerships, processes, learning environment, and resources. As service learning placements are complex in nature and encompass stakeholders, partnerships, learning environment, and resources, both frameworks were used in this study (Table 1). Future research could explore development of a suitable framework to progress the theory and evidence building for effective service learning placements.

People including the right supervisee, right supervisor, university staff, and community members with the right expectations are shown in this study as crucial to the success of service learning placements. A previous rural Australian study concerning blended supervision of junior doctors supports this and illuminates the importance of having the right supervisor and right supervisee in the right work context [19]. Findings from the current study also highlight that innovative models of supervision in rural communities are not for everyone and may be best reserved for learners that are motivated to learn, willing and open to try new experiences, and with a genuine interest in rural health. Placement coordination staff in universities can be educated on the importance of finding the right student match for these placements.

Several respondents noted the use of interprofessional supervision, peer supervision, and telesupervision models. Interprofessional education and practice principles have been previously successfully incorporated into service learning placements. Studies have documented the benefits of this approach within allied health disciplines [3, 20]. Interprofessional learning and practice reflective of true teams providing holistic and seamless care involve collaboration not only within allied health, but also with other professions including nursing, and medicine [21]. There is a need to extend inter-discipline, inter-faculty, and inter-sectoral collaborations to provide more ‘real-world’ authentic learning experiences to students [4]. While respondents noted the use of telesupervision as part of their attempts to maintain service learning placements during COVID-19 restrictions, further information is unavailable on the technology used or user experience and satisfaction with this. Telesupervision can only be effective if set up well [22]. Further studies can explore these non-traditional and emerging models of supervision within the service learning context.

**Fig. 1:**
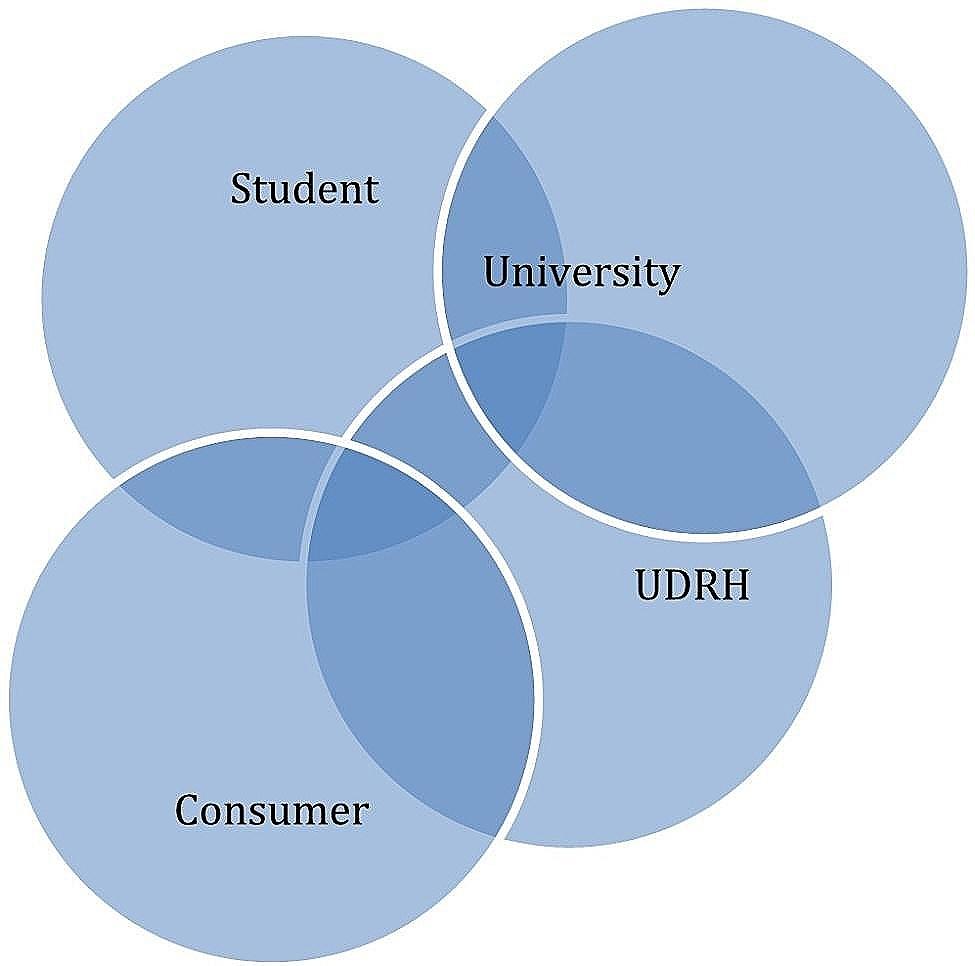
Relationships between Service Learning Stakeholders of UDRHs

Study findings indicate that relationships and partnerships between all parties are crucial enablers of successful service learning placements. In the early service learning examples from North American universities, the university is positioned as a citizen collaborating directly with its community, embedding students for the benefit of both the community and students. The current study points to a gap in communication between parent universities, UDRH staff, and communities that may be detrimental to service learning, and ultimately to the provision of services in rural communities. The slow evolution of UDRHs and their established roles and locations within rural communities has resulted in strong relationships with local communities and consumers. This allows UDRH educators and clinical supervisors to negotiate and develop bespoke service learning opportunities with community partners, and work with universities to match appropriate students. Students completing placements with UDRHs are exposed to relationships, negotiations, and engagements with their own universities when preparing for placement, and with the UDRH and community members during their service Iearning placements. Communities and consumers engage with UDRHs and students through their participation in the planning, delivery, and evaluation of service learning programs. The parent universities engage predominantly with their students and with the UDRHs, but findings suggest that they do not always have deep and enduring relationships with rural communities, and consumers. Overlapping relationships and relationship gaps identified in this study are displayed in the Venn diagram (Fig. 1).

Relationship gaps in service learning are a significant challenge that needs to be acknowledged and addressed. This can enable universities to have more authentic and enduring relationships and demonstrate commitment to engaging with rural communities, and to prepare students for rural placements including intentional service learning opportunities. Successful service learning placements, in addition to providing learning opportunities for students and meeting a service need in the community [2, 3, 20, 23], can also influence future recruitment of the healthcare workforce [15, 24]. This can be an impetus for all involved stakeholders to further collaborate and enhance partnerships. Further case studies of UDRHs that have had success in engagement with all partners, along with information already available on facilitating successful partnerships [23] will be useful in providing a roadmap for established and newer UDRHs involved in facilitating service learning.

The impact of the COVID-19 pandemic on provision of service learning placements was noted by some respondents in the context of maintaining students in place in rural and remote communities. Campbell and colleagues [2] outline several short, medium, and longer term responses adopted to sustain several service learning placements in remote northern Australia since the pandemic onset. These strategies largely capitalize on the utilization of technology, stakeholder flexibility, and the role of quality improvement projects in ensuring learning opportunities [2]. These are consistent with findings from the current study. Evaluation of these strategies are needed to inform sustainability of service learning placements into the post-pandemic period.

### Implications for practice, policy, and research

This study provides evidence on the usefulness of service learning placement models not only to facilitate student learning but also to address service delivery needs in under-resourced rural and remote communities in Australia. Appropriate resources, infrastructure, staffing, and funding to initiate, support, and sustain service learning placements are crucial. This study provides information on the need for funding and support that can be considered by policy makers, grant funding bodies, and philanthropic organisations. Future research can investigate the role of service learning placements from student, consumer, and community perspectives, and can utilize in-depth qualitative methods. There is also a scope for the development of a tailored framework to progress this area.

### Strengths and limitations

This national study with a high response rate utilized data from all Australian states to obtain a comprehensive snapshot of how service learning placements are being used. Utilization of a mixed methods hybrid analysis approach enabled close alignment to existing theory, without losing the opportunity to also learn from the data. Although the survey was designed to collect both numerical and textual data, and rich data were obtained, a further follow-up study utilizing a more in-depth qualitative design can further help to build evidence on the contextual factors that play a role in this complex field. This study only surveyed UDRH staff perspectives which could be biased. It is also limited by the absence of student and community members’ experiences.

## Conclusion

This national study investigated how service learning placements are being implemented across rural and remote Australia and has shed light on factors that enable or hinder the utilization of such programs from the perspective of the UDRHs who facilitate these programs. Using existing international frameworks to inform data analysis and interpretation of findings, this study adds high-quality evidence to a sparsely researched field and extends our understanding of the barriers and enablers for such programs based on available data. Further research is needed to investigate student, consumer, and community experiences and outcomes of service learning placements. The results of this study also provide a launch point for the development of a model to underpin service learning programs across Australia through future research.

### Electronic supplementary material

Below is the link to the electronic supplementary material.

Supplementary Material 1

## Data Availability

All data are protected by ethics. Reasonable requests to access de-identified data can be made to the first author and is subject to ethics approval.
