# Supplementary material for: Australian rural service learning student placements: a national survey
Source: BMC Med Educ. 2024 Mar 1;24:216. doi: 10.1186/s12909-024-05172-0 (PMC10908018; doi:10.1186/s12909-024-05172-0)
Supplement: Supplementary file 1 — Supplementary Material 1 [file 12909_2024_5172_MOESM1_ESM.docx]

**Supporting Information – Survey**

1. What is the name of your UDRH?
2. How many years has your UDRH been completing service learning placements?

- Service learning
- Work ready
- Role emerging
- Project placements
- Other

1. What terms does your UDRH use for service learning placements, that could be related/similar? e.g. role emerging, non-traditional etc.
2. What percentage of your placement weeks are service learning placements?
3. What kinds of service-learning placement models does your UDRH facilitate?

- Student-led clinic
- Organisational based (Schools, aged care facilities etc.)
- Project based
- Research based
- Aboriginal Community controlled Health Organisations (ACCHOS)
- Outreach service
- Other

1. Describe the supervision models that you utilise in your service learning placements. e.g. remote, interprofessional, external, internal, peer, near-peer, long-arm etc
2. Which health disciplines participate in your service learning placements? (Please select all that apply).

- Physiotherapy
- Speech pathology
- Occupational therapy
- Nutrition and Dietetics
- Exercise Physiology
- Audiology
- Chiropractic
- Oral Health
- Music Therapy
- Social Work
- Nursing
- Podiatry
- Medical Imaging
- Optometry
- Dentistry
- Other

Please specify other disciplines

1. Where do students complete their service-learning placements? Please indicate for each option if you have service learning placements in these settings, and if so, which Modified Monash categories or categories these are located within.

NOTE: If you do not have service learning placements in a listed setting, please select ‘Not Applicable’.

| **Setting** | **Not Applicable** | **MM2** | **MM3** | **MM4** | **MM5** | **MM6** | **MM7** |
| --- | --- | --- | --- | --- | --- | --- | --- |
| Primary Schools |  |  |  |  |  |  |  |
| Secondary Schools |  |  |  |  |  |  |  |
| Early Childhood Centres |  |  |  |  |  |  |  |
| Aboriginal Community Controlled Health Organisations (ACCHOS) |  |  |  |  |  |  |  |
| Outpatients (where funding is not sufficient or help with waitlists) |  |  |  |  |  |  |  |
| Home visits (where funding is not sufficient or help with waitlists) |  |  |  |  |  |  |  |
| Pop up clinics |  |  |  |  |  |  |  |
| Neighbourhood and community centres |  |  |  |  |  |  |  |
| Small health services |  |  |  |  |  |  |  |
| Not for profit organisations |  |  |  |  |  |  |  |
| Private practices |  |  |  |  |  |  |  |
| Residential aged care settings |  |  |  |  |  |  |  |

If your service learning placements are located in other settings, please specify and indicate for each the MM categories of the towns these are located in

**Service learning Governance Structures**

1. Do you have governance structures in place for your service learning placements? E.g. a committee or working group

- Yes
- No

1. What are the key factors that have contributed to successful service learning placements? Please separate these into those for a) students, b)communities, c) universities, and d) the UDRH, and enter them into the relevant sections below.

| 10 a | For students (3 main factors) |  |
| --- | --- | --- |
| 10 b | For communities (3 main factors) |  |
| 10 c | For universities (3 main factors) |  |
| 10 d | For UDRH (3 main factors) |  |

1. What challenges have you faced in coordinating service learning placements? What strategies helped solve them? What hasn’t worked? Please separate these into those for a) students, b)communities, c) universities, and d) the UDRH, and enter them into the relevant sections below.

| 11 a | For students (3 main factors) |  |
| --- | --- | --- |
| 11 b | For communities (3 main factors) |  |
| 11 c | For universities (3 main factors) |  |
| 11 d | For UDRH (3 main factors) |  |

**Service learning research and evaluation**

1. Are you researching/evaluating your service learning models?

- Yes
- No

1. How many people contributed to this survey?
